# The usefulness of serum procalcitonin levels in predicting surgical intervention in patients with tubo-ovarian abscess

**DOI:** 10.1590/1806-9282.20241294

**Published:** 2025-05-02

**Authors:** Osman Samet Gunkaya, Ayşegül Bestel

**Affiliations:** 1 University of Health Sciences Turkey, Sancaktepe Sehit Prof. Dr. Ilhan Varank Training and Research Hospital, Department of Obstetrics and Gynecology – İstanbul, Turkey.; 2 University of Health Sciences Turkey, Kanuni Sultan Suleyman Training and Research Hospital, Department of Obstetrics and Gynecology, İstanbul, Turkey.

**Keywords:** Abscess, Biochemical markers, Pelvic inflammatory disease, Patient selection, Procalcitonin

## Abstract

**OBJECTIVE:**

Pelvic inflammatory disease is one of the most common gynecological diseases, and 15% of cases are accompanied by tubal-ovarian abscesses. The aim of this study was to evaluate the usefulness of abscess mass size, serum procalcitonin, and other biochemical markers in patients with tubo-ovarian abscesses in predicting surgical intervention.

**METHODS:**

This case–control study included 113 women who were diagnosed with tubo-ovarian abscess, hospitalized, and started on antibiotic treatment. Demographic characteristics, biochemical markers, ultrasound findings, and length of hospital stay were recorded during medical treatment.

**RESULT:**

In terms of demographic characteristics, there was no significant difference between cases requiring complete recovery with medical treatment and cases requiring surgery for complete recovery. While serum cancer antigen 125 level was not statistically significant, there was a significant difference among biochemical markers: serum white blood cell level (18,007.0±6,406.3; p=0.001), C-reactive protein level (261.2±122.2; p<0.001), procalcitonin level (0.88±0.46; p<0.001), and abscess mass size (6.1±1.2; p<0.001) in cases that required surgery for full recovery. The highest sensitivity variable predicting surgical intervention in tubo-ovarian abscess patients was abscess mass size (cut-off value>5.25 cm and area under the curve 0.768) with a sensitivity of 72.2%. The second highest sensitivity variant, procalcitonin (cut-off value>0.635 ng/mL and area under the curve 0.756), showed a sensitivity of 70.4%.

**CONCLUSION:**

Although procalcitonin provides information about the severity of the disease in patients with tubo-ovarian abscesses, evaluation of the abscess mass size along with its size was useful in deciding surgical intervention.

## INTRODUCTION

A tubo-ovarian abscess (TOA) is an inflammatory mass that affects the ovary, fallopian tube, peritoneal tissue, and sometimes other nearby pelvic organs, including the bladder or bowel, and TOAs are often a consequence of pelvic inflammatory disease (PID)^1^. PID risk factors include having first sexual intercourse under the age of 25, having multiple sexual partners, using an intrauterine device, and having a low socioeconomic status^2,3^. While the diagnosis of PID is made by physical examination, some ultrasonographic classifications such as unilocular cystic structure, complex multicystic appearance, and pyosalpinx have been created to define the sonographic appearance of TOA^4^. Diagnosis is often difficult due to nonspecific symptoms and findings, and delayed diagnosis may cause TOAs to rupture, leading to life-threatening sepsis cases^5^. In addition, TOA often affects women of childbearing age and causes infertility, ectopic pregnancies, pelvic adhesions, and chronic pelvic pain^1^. The first treatment option for TOA is broad-spectrum parenteral antibiotic therapy. However, 25–30% of cases do not respond to medical treatment and require surgical intervention^6,7^. Advanced age, high white blood cell count (WBC), and cysts larger than 6 cm were found to be factors that lead to medical treatment failure in cases of TOA^8^. Although abdominal examination, fever measurement, and blood count are the basic components in the follow-up of the disease, it is known that the measurement of C-reactive protein (CRP) and serum procalcitonin (PCT) in the diagnosis and treatment follow-up gives information about the severity of the disease^9^.

The aim of our study was to evaluate serum procalcitonin levels, other laboratory parameters, and abscess mass size in patients hospitalized with a diagnosis of TOA and investigate whether these parameters are associated with medical treatment failure and, therefore, whether they are effective in predicting surgical intervention.

## METHODS

### Patients

We conducted this case–control study in patients hospitalized for TOA and started medical treatment in the gynecology department of a tertiary medical center between January 2019 and October 2023. Patients with a pelvic mass on ultrasonography diagnosed with TOA based on anamnesis, laboratory, and physical examination findings were included in the study. Pelvic inflammatory mass was evaluated according to the suggestions of the US Centers for Disease Control and Prevention criteria^10^.

### Data collection

The study was conducted with the institutional ethics committee (KAEK/2023.06.68) and in line with the Declaration of Helsinki. All patients gave written informed consent before enrolling in the study. TOA was diagnosed in patients presenting with lower abdominal and pelvic pain, vaginal discharge, and cervical motion tenderness during gynecological examination. Patients with incomplete data were not included in the study. Demographic data of all patients, laboratory parameters, pelvic ultrasonography findings, treatment method, and the length of hospital stay were recorded. All cases underwent an ultrasonography evaluation using a 6–10-MHz transvaginal probe (LOGIQ P5; GE Healthcare Inc., Milwaukee, WI). At sonographic examination, maximum diameters were recorded. Venous blood samples were obtained on admission to analyze serum WBC counts, CRP, and procalcitonin levels. Complete blood count was assessed with a cell counter (Abbott, Cell Dyn 3700, Abbott Diagnostics, 2010). Serum CRP concentrations were used as per standard methodology (Abbott, Acrhitecti1000sr, Abbott Diagnostics, 2012). Serum procalcitonin levels of the cases were analyzed (Wondfo, Finecare Multichannel, China, 2012).

### Treatments

Hospitalization and intravenous antibiotics are suggested for all patients diagnosed with TOA. The antibiotic regimen included ceftriaxone 1.0 g IV every 12 h, metronidazole 500 mg IV every 8 h, and isepamicin 400 mg IV every 24 h. Surgical intervention was considered in cases of acute clinical deterioration such as persistent fever, sepsis, enlarging TOA, worsening pelvic tenderness, ruptured abscess, or widespread peritonitis despite 72 h of antibiotic treatment. Postoperative antibiotics were continued until clinical improvement was achieved. The surgical technique was determined according to the surgical exploration findings (drainage, uni/bilateral salpingectomy/salpingo-oophorectomy, or total abdominal hysterectomy combined with uni/bilateral salpingectomy/salpingo-oophorectomy).

### Statistics

Ultrasonographic findings, demographic and laboratory parameters of women who had completely recovered with medical treatment, and women who had undergone surgical treatment were compared. All analyses were performed with the SPSS 20 program package (SPSS Inc., Chicago, IL). Mean and standard deviation were used for descriptive statistics. The characteristics of the two groups were compared using independent t-test and the chi-square test. The correlations were assessed using Spearman's correlation coefficient, along with the related p-values. A binary logistic regression was performed for significant variables in the univariate analysis. All statistical tests were two-sided, and a p-value of <0.05 was considered significant. A receiver operating characteristic (ROC) curve was used to determine the cut-off of clinical characteristics about the need for surgical intervention. The 95% confidence interval (CI) of the area under the curve (AUC) was determined. The optimum diagnostic threshold was chosen by using the ROC curve. The sensitivity, specificity, accuracy, positive predictive value (PPV), negative predictive value (NPV), and odds ratio of significant variables were calculated to predict the risk of surgical intervention in patients with TOA. Significant variables in the multivariate analysis were also combined to maximize the predictability of surgical intervention.

## RESULTS

During the study period, 243 people were diagnosed with PID. Of these, 113 patients received a final diagnosis of TOA. Of the patients, 58 were treated with intravenous antibiotics (in the conservative treatment group) and 55 did not respond to medical treatment and required further surgery or abscess drainage (surgical intervention group). Table 1 shows the patient demographic and clinical data for the two groups. While there was no significant difference between demographic characteristics, the mean (standard deviation) of biochemical markers WBC count, PCT, CRP, mass size, and hospital stay were higher in the surgical intervention group than in the conservative treatment group: 18,007.0 (6,406.3) μL and 14,669.3 (4,675.8) μL, p=0.001; 0.88 (0.46) ng/mL vs. 0.52 (0.28) ng/mL, p<0.001; 261.2 (122.2) mg/L vs. 184.2 (88.4) mg/L, p<0.001; 6.1 (1.2) cm vs. 4.9 (1.0) cm, p<0.001; and 11.4 (4.1) days vs. 8.0 (3) days, p<0.001, respectively.

**Table 1:** Comparison of some demographic and clinical characteristics between cases who responded and failed to respond to medical treatment.

	Full recovery with medical treatment (n=58)	Cases that required surgery for full recovery (n=55)	p-value
Age (year)	37.6±6.5	38.0±5.4	0.688^1^
Parity; nulliparous, n (%)	10 (17.2)	4 (7.3)	0.108^2^
BMI (kg/m^2^)	26.4±4.9	27.2±3.8	0.329^1^
Previous cesarean section, n (%)	13 (22.4)	19 (34.5)	0.153^2^
Use of IUD, n (%)	29 (50)	27 (49.1)	0.923^2^
Smoking, n (%)	3 (5.2)	2 (3.6)	0.691^2^
Length of hospital stay (days)	8.0±3	11.4±4.1	***<0.001*** 1
Mass size (cm)	4.9±1.0	6.1±1.2	***<0.001*** 1
WBC count (uL)	14,669.3±4,675.8	18,007.0±6,406.3	***0.001*** 1
CRP (mg/L)	184.2±88.4	261.2±122.2	***<0.001*** 1
PCT (ng/mL)	0.52±0.28	0.88±0.46	***<0.001*** 1
CA-125 (Unit/mL)	58.4±43.2	56.6±33.5	0.808^2^

1 Independent t-test.

2 Chi-square test.

BMI: body mass index; IUD: intrauterine device; WBC: white blood cell; CRP: C-reactive protein; PCT: procalcitonin; CA-125: cancer antigen 125. Statistically significant values are indicated in bold. Italicized p-values indicate statistical significance (p<0.05).

In the 55 surgical intervention group patients, laparoscopy and laparotomy were performed in 34 (61.8%) and 21 (38.2%) patients, respectively. In total, 41 patients underwent unilateral salpingectomy or unilateral salpingo-oophorectomy, and 10 patients underwent bilateral salpingectomy or bilateral salpingo-oophorectomy. Notably, four cases were identified who underwent total hysterectomy in addition to adnexal surgery. An appendectomy was performed in one patient because TOA had spread to the periappendix area.

Univariate analysis was performed using binary logistic regression. As shown in Table 2, three parameters remained significantly associated with the risk of surgical intervention: mass size (OR 2.951; 95%CI 1.665–5.229); WBC (OR 1.000; 95%CI 1.000–1.000), and PCT (OR 29.376; 95%CI 5.360–160.996). CRP showed no significance (p=0.806). In addition, no correlation was found between serum procalcitonin levels and WBC (r=-0.118, p=0.392, r: Pearson's rank correlation) and mass size (r=-0.083, p=0.392, r: Pearson's rank correlation), while a positive correlation was found between CRP levels (r=0.283, p=0.036, r: Pearson's rank correlation). The highest sensitivity variable predicting surgical intervention in TOA patients was abscess mass size (cut-off value>5.25 cm and AUC 0.768) with a sensitivity of 72.2%. The second highest sensitivity variant, PCT (cut-off value>0.635 ng/mL and AUC 0.756), showed sensitivity 70.4% (Table 3).

**Table 2:** Binary regression analysis for surgery management of the patient with tubo-ovarian abscess.

	OR	95%CI	p-value
Mass size (cm)	2.951	1.665–5.229	***<0.001***
WBC (μL)	1.000	1.000–1.000	***0.030***
CRP (mg/dL)	1.001	0.995–1.006	**0.806**
PCT (ng/mL)	29.376	5.360–160.996	***<0.001***

OR: odds ratio; CI: confidence interval; WBC: white blood cell; CRP: C-reactive protein; PCT: procalcitonin. Statistically significant values are indicated in bold. Italicized p-values indicate statistical significance (p<0.05).

**Table 3:** Evaluation of the efficacy of mass size, white blood cell, C-reactive protein, and procalcitonin in need of surgery intervention.

	Cut value	AUC (95%)	p-value	Sensitivity (%)	Specificity (%)
Mass size (cm)	5.25	0.768 (0.683–0.854)	**<0.001**	72.2	69
WBC (μL)	21,660	0.640 (0.537–0.742)	**0.011**	27.8	96.6
CRP (mg/dL)	313.5	0.679 (0.581–0.777)	**0.001**	31.5	96.6
PCT (ng/mL)	0.635	0.756 (0.666–0.846)	**<0.001**	70.4	72.4

WBC: white blood cell; CRP: C-reactive protein; PCT: procalcitonin; AUC: area under the curve. Statistically significant values are indicated in bold.

Mass size, WBC, CRP, and PCT were analyzed based on ROC curves as determinants of TOA. Sensitivity, specificity, accuracy, PPV, and NPV were calculated using the cut-off values. As a single variable, mass size (cut-off value>5.25 years and AUC 0.768) showed the highest sensitivity (72.2%) for predicting surgical intervention in TOA patients. However, the specificity of mass size was only 69%. The second highest sensitivity variable, PCT (cut-off value>0.635 ng/mL and AUC 0.756), showed sensitivity (70.4%) for predicting surgical intervention in TOA patients. Other parameters are shown in Table 3. To increase predictability, patients with mass sizes greater than the cut-off and PCT levels higher than the cut-off value were combined. The sensitivity of this group combination index was 92.7%, specificity 46.5%, accuracy 84.5%, PPV 62.1%, and NPV 87%.

## DISCUSSION

Early identification of the risk of antibiotic treatment failure in TOA may alert clinicians to the need for changes in treatment strategy, and early surgical intervention reduces acute and long-term complications of TOA^11,12^. Therefore, inflammatory markers and mass size can help the doctor focus his or her attention on people who will require surgical intervention. This study found that although mass size has the highest sensitivity in TOA, evaluation of mass size and PCT level together can predict the need for surgical intervention in TOA with 92.7% sensitivity.

Previous studies reported that CRP and WBC levels helped predict antibiotic treatment failure in TOA patients treated with antibiotics^13^. Nevertheless, according to another study, CRP levels did not differ between TOA patients who responded to antibiotic treatment and those who did not^8^. In our study, there was a significant difference in CRP levels between the TOA patient group that responded to antibiotics and the patient group that did not respond and required surgery, but its sensitivity in predicting the need for surgery was low (31.5%).

There are studies in the literature showing that high CA-125 serum levels are associated with the failure of parenteral antibiotic treatment in TOA^13^. In our study, it was found that serum Ca 125 levels did not have a significant value in surgical prediction.

Previous studies have shown that abscess size increases surgical treatment rates in TOA^14^. Similarly, it has been found that TOA abscess sizes over 8 cm increase the need for surgical intervention and the duration of hospitalization^15^. In another study, TOA abscess size greater than 6.5 cm was predictive of patients requiring surgical treatment^16^. In our study, the cut-off value for predicting the need for surgical intervention was found to be 5.25 cm, and the sensitivity was 72.2%. This mass size value was slightly lower than those in the literature.

When diagnosing sepsis and systemic infection, PCT is a helpful sign. Furthermore, a study addressing the usefulness of PCT in identifying TOA in female PID patients exists^9^. Nevertheless, there is little evidence in the literature regarding the PCT level's predictive power for surgical intervention in patients with TOA. In our study, the PCT cut-off value was calculated for predicting surgical intervention. This value was 0.636 ng/mL, and its sensitivity was 70.4%. This PCT value was found to be the parameter with the highest sensitivity after abscess size in predicting surgical intervention. We believe that when TOA mass size and serum procalcitonin level are evaluated together in TOA patients, they can be used to predict surgical treatment, which will provide additional advantages in terms of cost, and late complications can be prevented.

This study had some limitations. First, this is a retrospective design, and the sample size is relatively small. Second, because procalcitonin begins to increase markedly 8–12 h after illness onset, it may not accurately reflect clinical severity in patients hospitalized in the early stages of PID. Another limitation is that the diagnosis of TOA is made clinically. Finally, the lack of culture and antibiotic sensitivity test findings was one of the drawbacks, and all patients were treated with empirical antibiotics. The strength of the study is that, to the best of our knowledge, it is the first study to evaluate the prediction of surgical intervention PCT in TOA patients.

In conclusion, although PCT provides information about the severity of the disease in patients with TOA, its evaluation together with the size of the abscess mass was found to be more sensitive in deciding on surgical intervention and could be easily applied in clinical practice. Nowadays, inflammatory ratios (such as the SII) are becoming increasingly important in obstetrics and gynecology^17^. Therefore, randomized controlled trials with these ratios and procalcitonin could be conducted in the future.

## Data Availability

The data that support the findings of this study are available from the corresponding author upon reasonable request.
